# Dexamethasone-Sparing Antiemetic Prophylaxis for Chemotherapy-Induced Nausea and Vomiting in Highly and Moderately Emetogenic Chemotherapy: The SHEILD Study

**DOI:** 10.7759/cureus.70290

**Published:** 2024-09-26

**Authors:** Sudheer Reddy, Suresh B Kumar, Tirumala Venkatesh, Uday Kumar Punukollu, Suyash B Sharma, Richa Tripathi

**Affiliations:** 1 Oncology, Omega Cancer Hospital, Kurnool, IND; 2 Medical Oncology, Kauvery Hospital, Chennai, IND; 3 Oncology, DBR and SK Super Specialty Hospital and Cancer Center, Tirupati, IND; 4 Oncology, Renova Century Hospital, Telangana, IND; 5 Medical Affairs, Zydus Lifesciences Ltd., Ahmedabad, IND

**Keywords:** cinv, dexamethasone, highly emetogenic chemotherapy, moderately emetogenic chemotherapy, nepa

## Abstract

Background: Chemotherapy-induced nausea and vomiting (CINV) significantly impacts patient‘s quality of life and treatment adherence. This study investigated the efficacy of Generic Netupitant and Palonosetron tablets (Nykron) with dexamethasone single dose for CINV prophylaxis in patients receiving highly emetogenic chemotherapy (HEC) and moderately emetogenic chemotherapy (MEC). Additionally, this approach aligns with the principles of the SHIELD study (Sparing High Efficacy Intervention for Low Dose Dexamethasone), which focuses on maximizing antiemetic effectiveness while minimizing dexamethasone use.

Methodology: This multicenter retrospective study evaluates data from patients who received HEC/MEC and were administered a fixed-dose combination of Generic NEPA (Netupitant 300 mg and Palonosetron 0.5 mg tablets, Nykron combi-pack) along with a single dose of dexamethasone (12 mg/8 mg) before chemotherapy. The data were collected from September 2022 till September 2023. Outcomes measured included complete response (no vomiting and no need for rescue medications), complete protection (no significant nausea (<2.5 cm on VAS), no vomiting, and no use of rescue medication), and complete control (no emetic episodes, no rescue therapy, and no nausea [0 cm on VAS]) during the acute phase (0-24 hours) and delayed phase (24-120 hours) post-chemotherapy.

Results: The data of 372 patients was evaluated in which breast cancer was the most common cancer with 223 (59.95%) patients for which doxorubicin and cyclophosphamide (192, 51.61%) was the most administered chemotherapy combination. The second most common cancer was gastrointestinal (GI) cancer with stomach cancer in 47 (12.6%), colorectal cancer in 4 (1%), and pancreatic cancer in 2 (0.54%). A total of 360 (96.8%) patients received an HEC regimen across the cycle, while only 5 (1.3%) received an MEC regimen.

The regimen demonstrated exceptional efficacy with a 96.9% overall response rate across all cycles. Complete control rates for acute CINV were 92% and 90% for delayed CINV across chemotherapy cycles. Complete response rates remained consistently high (94%-98%) across all cycles and overall phases. Only 3% of patients experienced anticipatory CINV.

Conclusions: This dexamethasone-sparing Generic NEPA regimen showed remarkable efficacy in CINV management for HEC/MEC regimen-receiving patients, maintaining high response rates in both acute and delayed across all cycles. These findings indicate a potential paradigm shift in CINV prophylaxis, necessitating further investigation through prospective, randomized controlled trials to validate long-term safety and efficacy.

## Introduction

Cancer poses a significant health challenge globally, and the statistics reflect a growing burden. In 2022, approximately 20 million new cancer cases were diagnosed worldwide, leading to 9.7 million cancer-related deaths. In India, there is an anticipated 12.8% rise in incidence by 2025 compared to 2020. In 2022 alone, there were 1.3 million new cases and 850,000 cancer-related deaths [1]. Chemotherapy remains a cornerstone of cancer treatment, yet it frequently results in substantial adverse effects, including myelosuppression, cardiotoxicity, and nephrotoxicity. Notably, chemotherapy-induced nausea and vomiting (CINV) affects approximately 40% of cancer patients in India [2].

CINV significantly impairs the patient's quality of life and adherence to treatment regimens, encompassing acute, delayed, anticipatory, and breakthrough/refractory forms [2]. The management of CINV in India is further challenged by barriers such as limited access to effective antiemetics, variable healthcare infrastructure, and economic constraints [3]. Traditional treatments, including 5-hydroxytryptamine 3 (5-HT3) receptor antagonists and corticosteroids, often fall short in both efficacy and patient adherence. While traditional treatments like 5-HT3 receptor antagonists and corticosteroids are widely used, they often fail to fully manage CINV due to limited efficacy, patient adherence issues, and side effects, whereas advanced therapies like chimeric antigen receptor (CAR) T-cell therapy show promise but require further evaluation in this context.

Addressing the urgent need for improved CINV management strategies in India, NEPA, an oral fixed-dose combination of netupitant and palonosetron, emerges as a promising therapeutic option. Netupitant, a highly selective NK1 receptor antagonist, targets delayed emesis by preventing the binding of substance P, while palonosetron, a second-generation 5-HT3 receptor antagonist, blocks serotonin to combat acute emesis. This dual-action mechanism effectively targets both acute and delayed phases of CINV, providing superior control compared to existing treatments by simultaneously inhibiting two critical pathways involved in the emetic response [4,5].

Current treatments often fail to control delayed emesis, leading to prolonged nausea and vomiting. Adherence to CINV guidelines is inconsistent due to cost, accessibility, and awareness issues, and single-agent therapies are often insufficient, complicating treatment regimens [5]. NEPA’s dual-action approach ensures continuous protection against CINV, simplifies treatment, improves patient adherence, and enhances quality of life, particularly in resource-limited settings like India [6].

NEPA holds significant potential to enhance CINV control, improve patient adherence to chemotherapy, and offer the convenience of a single oral dose [6]. This retrospective, multicenter study aims to evaluate the efficacy of NEPA in managing CINV among Indian patients undergoing highly emetogenic chemotherapy (HEC). By analyzing data from a diverse patient population across multiple centers, this study seeks to provide robust evidence of low-dose dexamethasone and NEPA's clinical benefits. Moreover, the study aligns with the principles of the SHIELD (Sparing High Efficacy Intervention for Low Dose Dexamethasone) framework, emphasizing an optimized approach that maximizes antiemetic efficacy while minimizing dexamethasone use, ultimately improving both patient outcomes and treatment adherence.

## Materials and methods

Study design

This retrospective, multicenter, open-label, single-arm, investigator-initiated study analyzed the data of 372 patients treated with HEC and moderately emetogenic chemotherapy (MEC). Study data were collected from different centers in Hyderabad, Chennai, and Tirupati from September 2022 till September 2023. Ethical approval was obtained for data analysis from the ACEAS Ethics Committee (EC; EC number: TV/02/07/23).

Participants

The data of adult patients of both sexes, aged 18 years and older, who were undergoing HEC and MEC as per National Comprehensive Cancer Network (NCCN) 2023 Antiemetic guidelines, were included. The study also included data from patients with confirmed cancer diagnosis through histological or cytological examination and an Eastern Cooperative Oncology Group (ECOG) performance status of 0 to 2. Patients’ data with acceptable hepatic function (transaminase levels not exceedingly twice the upper limit of normal), and renal function (creatinine levels below 1.5 times the upper limit of normal), were included to avoid other causes of nausea and vomiting skewing the results.

Data of patients who were pregnant or lactating or had a history of myocardial infarction within the past six months or had documented hypersensitivity to 5-HT3 receptor antagonists (5-HT3RA) or neurokinin-1 receptor antagonists (NK1RA) or their excipients were excluded. Additional exclusion criteria included uncontrolled diabetes mellitus, baseline nausea and vomiting, gastrointestinal obstruction, or active peptic ulcer disease.

Study objectives

The primary outcome was the treatment response, defined by the absence of nausea and emesis during both the acute phase (0-24 hours) and delayed phase (24-120 hours) following chemotherapy administration. Secondary outcomes included complete response (no vomiting and no need for rescue medications) and complete control (no emetic episodes, no rescue therapy, and no nausea [0 cm on VAS]), complete protection (no significant nausea [<2.5 cm on VAS], no vomiting, and no use of rescue medication) during the acute phase (0-24 hours) and delayed phase (24-120 hours) post-chemotherapy. Overall complete response (CR-O), defined as no vomiting and no need for rescue medication, at cycle 1 (time frame: 0-120 hours) [6]. Overall complete protection (CP-O), defined as no significant nausea (<2.5 cm on VAS), no vomiting, and no use of rescue medication in the time frame of 0-120 hours.

Data collection and statistical analysis

Data were collected retrospectively from patient records at the participating centers. Treatment response and other variables were assessed through clinical evaluations and patient-reported outcomes during chemotherapy cycles. Groupings were chosen based on clinical relevance and predefined study objectives. All the data were collected and organized using Microsoft Excel version 2021 and continuous or quantitative variables were expressed as mean ± standard deviation (SD), and categorical variables were expressed as *n* (%).

Procedure

A triple oral combination of netupitant (300 mg), palonosetron (0.5 mg), and dexamethasone 12 mg/8 mg was administered one hour before chemotherapy initiation. Treatment response was evaluated during the acute (0-24 hours) and delayed (24-120 hours) phases.

## Results

The study evaluated the efficacy of a dexamethasone-sparing anti-emetic regimen for the prophylaxis of CINV in patients receiving HEC in India, with 299 (80.3%) being female as shown in Table 1. Many overlapping comorbidities are also considered, as shown in Table 1. Breast cancer was the most common indication, followed by stomach cancer and various other cancers with corresponding chemotherapy regimens as detailed in Table 2.

**Table 1:** Demographic profile of the study population. CINV, chemotherapy-induced nausea and vomiting; HBV, Hepatitis B virus

Variables			Values (*N *= 372)
Age (Mean ± SD)			54.23 ± 9.13
Gender, *n* (%)	Female		299 (80.38%)
	Male		73 (19.62%)
Comorbidities,* n *(%)		Hypertension	45 (12%)
		Diabetes	36 (9.6%)
		Coronary artery disease	02 (0.5%)
		HBV positive	01 (0.26%)
		Hypothyroidism	6 (1.61%)
Anticipatory CINV, *n *(%)			12 (3.23%)

**Table 2:** Patients receiving HEC and MEC chemotherapy for different cancer indications. HEC, highly emetogenic chemotherapy; MEC, moderately emetogenic chemotherapy

Indication	Total frequency	Chemotherapy received	Frequency *n* (%)
Breast Cancer	223	Doxorubicin and Cyclophosphamide	201 (90.13%)
		Epirubicin and Cyclophosphamide	6 (2.69%)
		Cisplatin	2 (0.90%)
		Cisplatin and Docetaxel	2 (0.90%)
		Docetaxel	1 (0.45%)
		Docetaxel and Cyclophosphamide	3 (1.35%)
		Docetaxel, Carboplatin, Pertuzumab, Trastuzumab, and Hyaluronidase	2 (0.90%)
		Gemcitabine and Carboplatin	1 (0.45%)
		Paclitaxel	1 (0.45%)
		Paclitaxel and Carboplatin	1 (0.45%)
		Paclitaxel, Carboplatin, and Pembrolizumab	2 (0.90%)
		Trastuzumab Emtansine	1 (0.45%)
Stomach cancer	47	FLOT regimen (Fluorouracil, Leucovorin, Oxaliplatin, and Docetaxel)	41 (87.23%)
		Docetaxel, Oxaliplatin, and tab Capecitabine	3 (6.38%)
		EOX (Epirubicin Oxaliplatin Capecitabine)	3 (6.38%)
Ovarian Cancer	31	Liposomal Doxorubicin Carboplatin	1 (3.23%)
		Paclitaxel and Carboplatin	30 (96.77%)
Head and Neck	17	Carboplatin and 5Fu (fluorouracil)	1 (5.88%)
		Cisplatin	8 (47.06%)
		Cisplatin and 5Fu (fluorouracil)	1 (5.88%)
		Cisplatin and Nimotozumab	1 (5.88%)
		Paclitaxel and Carboplatin	3 (17.65%)
		Pemetrexed and Carboplatin	1 (5.88%)
		TPF Chemo (Taxotere Cisplatin Fluorouracil)	2 (11.76%)
Soft tissue Sarcoma	17	Ifostomide and Doxorubicin	17 (100%)
Lung cancer	14	Cisplatin and Pemetrexed	1 (7.14%)
		Carboplatin	1 (7.14%)
		Cisplatin and Docetaxel	1 (7.14%)
		Cisplatin and Etoposide	1 (7.14%)
		High-dose Cisplatin	1 (7.14%)
		Paclitaxel and Carboplatin	2 (14.29%)
		Paclitaxel, Carboplatin, and Bevacizumab	2 (14.29%)
		Pemetrexed and Carboplatin	4 (28.57%)
		Pemetrexed, Carboplatin, and Zoledronic Acid	1 (7.14%)
		Pemetrexed, Carboplatin, and Bevacizumab	1 (7.14%)
Cervix	6	Paclitaxel and Carboplatin	2 (33.33%)
		Carboplatin-150	1 (16.67%)
Osteosarcoma	2	Cisplatin and Doxorubicin	2 (100%)
Pancreatic cancer	2	Gemcitabine and tab Capecitabine	1 (50%)
		Gemcitabine	1 (50%)
Prostate cancer	2	Gemcitabine and Cisplatin	1 (50%)
		Docetaxel, Zoledronic Acid, and Prednisolone	1 (50%)
Biliary tract cancer	1	Gemcitabine and Cisplatin	1 (100%)
Endometrium	1	Paclitaxel and Carboplatin	1 (100%)
Urinary bladder	1	Gemcitabine and Cisplatin	1 (100%)
Colorectal cancer	3	FOLFOX Q2W (fluorouracil, leucovorin, and oxaliplatin)	1 (33.33%)
		FOLFIRI (Folinic acid [leucovorin] Fluorouracil [5-FU] Irinotecan)	1 (33.33%)
		Bevacizumab & FOLFOX (fluorouracil, leucovorin, and oxaliplatin)	1 (33.33%)
Cutaneous SCC	1	Cisplatin	1 (100%)
Dysgerminoma	1	BEP (bleomycin, etoposide, and cisplatin)	1 (100%)
Neuro Endocrine Tumor	1	Epirubicin and Cyclophosphamide	1 (100%)
Oligodendroglioma	1	Bevacizumab and Irinotecan	1 (100%)
Unknown	1	Cisplatin	1 (100%)

**Figure 1:**
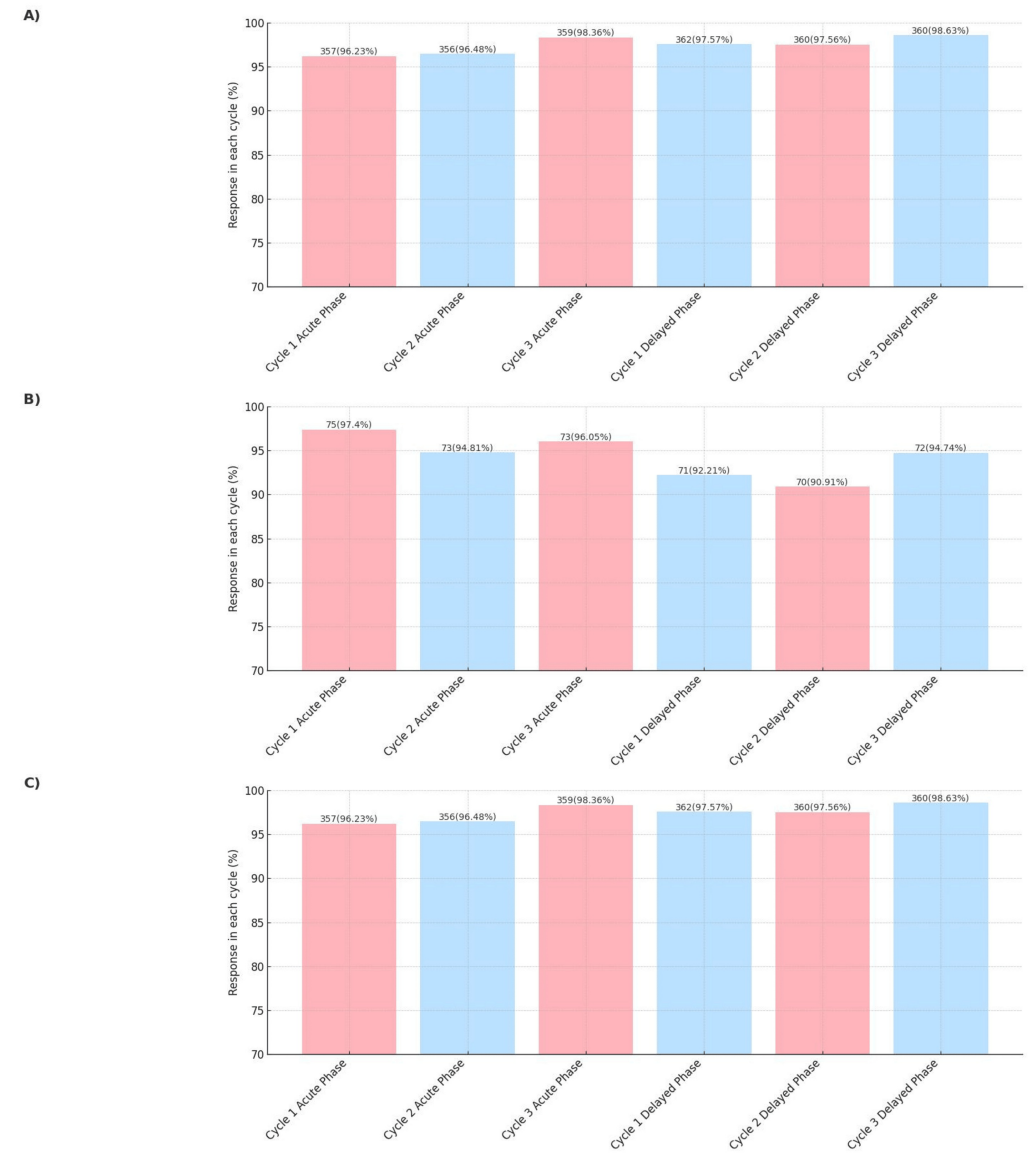
(A) Complete response, (B) complete protection, and (C) complete control of NEPA + Dex in chemotherapy-induced nausea and vomiting (CINV) patients across treatment cycles. NEPA, Netupitant 300 mg and Palonosetron 0.5 mg

Complete response rates were high in all cycles, ranging from 96% to 98% in the acute phase and 97% to 98% in the delayed phase (Figure 1A). For complete protection, response rates remained consistently high across all cycles, ranging from 94% to 97% in the acute phase and 90% to 97% in the delayed phase (Figure 1B). For complete control, the response rate remains consistent during all cycles (Figure 1C). During all cycles in the overall phase, the mean complete response, complete protection, and complete control in the overall phase were 97.02%, 94.34%, and 91.20%, respectively.

The treatment regimen maintained stable response rates for complete control of CINV symptoms in all the cycles, achieving 92% efficacy in the acute phase and 90% in the delayed phase. The study found that approximately 3% of patients experienced anticipatory CINV, underscoring the need for effective prophylactic strategies (Table 1).

The dexamethasone-sparing regimen demonstrated a high level of effectiveness, with an overall efficacy rate of 96.90% across all cycles and outcomes.

## Discussion

This study demonstrates the efficacy of NEPA in achieving effective CINV management, addressing a critical need for improved therapeutic options. A study of 372 Indian patients receiving HEC tested a dexamethasone-sparing antiemetic regimen (single-dose dexamethasone with NEPA). It showed high efficacy across four cycles, with complete control rates of 90%-92% in all three cycles. Complete response rates remained high (94%-98%) throughout all cycles.

The study's findings on the efficacy of a dexamethasone-sparing antiemetic regimen align well with existing research on NEPA for managing CINV [7-9]. The rationale for utilizing NEPA is underpinned by its dual mechanism of action, which targets both the acute and delayed phases of CINV. Hesketh et al. underscored the superiority of this dual-pathway inhibition compared to older 5-HT3 receptor antagonists, emphasizing NEPA's broad-spectrum antiemetic control [7].

The fixed-dose combination of NEPA simplifies administration, requiring only a single oral dose per chemotherapy cycle. This convenience is a significant advantage over more complex regimens, which often involve multiple doses and can lead to decreased patient adherence. Schwartzberg et al. highlighted that simpler regimens like NEPA's single-dose protocol enhance compliance, thereby improving overall treatment effectiveness [9]​​.

Furthermore, the study found that only 3% of patients experienced anticipatory CINV, a low incidence that underscores the effectiveness of the NEPA regimen in controlling acute and delayed CINV. Hesketh et al. also reported that NEPA was well-tolerated across diverse patient populations, including those with comorbid conditions such as hypertension and diabetes, similar to the present study [7]. Additionally, Poli-Bigelli et al. demonstrated that the addition of NK1 receptor antagonists to standard antiemetic therapy significantly improved the control of CINV in patients receiving HEC [8]​​. This supports the finding that NEPA, which includes netupitant, an NK1 receptor antagonist, effectively manages CINV.

The incorporation of a dexamethasone-sparing approach in the study is particularly noteworthy. Traditional antiemetic regimens often involve higher doses of dexamethasone, which can lead to adverse effects such as hyperglycemia, insomnia, and increased infection risk. By reducing the dexamethasone dosage to a single 0.5 mg dose before chemotherapy, the protocol potentially minimizes these side effects while maintaining high antiemetic efficacy. This approach aligns with the findings of Hesketh et al., who demonstrated that NEPA, even with reduced corticosteroid use, maintains robust antiemetic control [7]​.

Approximately 3% of patients experienced anticipatory CINV, emphasizing the importance of effective prophylactic strategies, as underscored by the American Society of Clinical Oncology (ASCO) and the National Comprehensive Cancer Network (NCCN) guidelines, which highlight the significance of prophylactic antiemetic therapy and combination regimens in minimizing the risk of CINV [10].

The dexamethasone-sparing regimen demonstrated a remarkable efficacy rate of 96.9% across all cycles, corroborating Schilling et al.'s findings on NEPA's superior ability to maintain quality of life and ensure safety in breast cancer patients undergoing AC chemotherapy. This underscores NEPA's significant real-world benefits and reinforces the necessity for personalized care in managing CINV [11]. The observed decline in efficacy during the fourth cycle underscores the imperative to investigate potential tolerance or adaptation mechanisms to antiemetic treatments [12]. These phenomena may stem from biological factors such as receptor desensitization or pharmacokinetic modifications due to repeated exposure [13]. Furthermore, patient-specific variables, including alterations in treatment regimen adherence, concurrent medications, or disease progression, could account for the variability in treatment response across chemotherapy cycles [14]. Addressing these factors through personalized medicine and ongoing treatment outcome monitoring could significantly enhance antiemetic strategies for patients receiving emetogenic chemotherapy.

The incidence of anticipatory CINV reveals the intricate relationship between psychological and physiological factors in chemotherapy patients [15]. Often initiated by conditioned responses to prior treatment cycles, anticipatory CINV highlights the critical need for comprehensive supportive care strategies. Behavioral interventions, cognitive-behavioral therapy, and patient education have demonstrated efficacy in reducing these symptoms and improving overall treatment adherence [16]. Integrating these approaches with potent pharmacological interventions like NEPA and dexamethasone provides a multifaceted strategy for managing CINV, aligning with current clinical guidelines that emphasize holistic patient care in oncology settings [17].

A study by Gralla et al. conducted a randomized, double-blind, placebo-controlled study that demonstrated the superior efficacy of NEPA compared to palonosetron alone in preventing CINV over multiple cycles of chemotherapy. Their findings showed that NEPA significantly improved complete response rates and quality of life among patients undergoing HEC [18]. Similarly, Aapro et al. in their Pan European Emesis Registry (PEER) study, evaluated the real-world impact of guideline-consistent antiemetic therapy, including NEPA, on CINV control. They found that adherence to guidelines significantly reduced the incidence of CINV, and NEPA's dual-action mechanism was particularly effective in both the acute and delayed phases of CINV [19].

While the retrospective design and lack of a control group necessitate cautious interpretation, the results are promising. Increasing the sample size in future studies could potentially provide a more robust understanding of the efficacy, though it's unlikely to drastically alter the positive trends observed. Further research, ideally through randomized controlled trials, is needed to confirm these findings and explore the long-term safety and efficacy of this regimen.

## Conclusions

This retrospective cohort study evaluated the efficacy of a dexamethasone-minimizing antiemetic regimen in Indian cancer patients over multiple chemotherapy cycles. The protocol, consisting of NEPA combined with a single dexamethasone dose, demonstrated effectiveness in controlling CINV. While the study design has inherent limitations, the results suggest clinical utility, particularly for patients with comorbidities such as hypertension and diabetes mellitus. The findings support further investigation of NEPA-based regimens in routine oncology practice. Additional prospective, randomized controlled trials are necessary to confirm these observations and potentially inform evidence-based guidelines for CINV management in diverse patient populations.
